# Systematic review and meta - analysis of risk prediction models for heart failure after PCI in patients with acute myocardial infarction

**DOI:** 10.1186/s12872-025-05406-z

**Published:** 2026-01-05

**Authors:** Xiongxiong Lu, Wen Ding, Jingyu Lu, Jingyao Wang, Bixin Wang

**Affiliations:** 1 General Hospital of Ningxia Medical University, Ningxia, China; 2 Ningxia Medical University, Ningxia, China

**Keywords:** Acute myocardial infarction, Percutaneous coronary intervention, Heart failure, Risk prediction model, Systematic review, Meta-analysis

## Abstract

**Background:**

The incidence of heart failure (HF) following percutaneous coronary intervention (PCI) in patients with acute myocardial infarction (AMI) remains relatively high, severely impairing long-term prognosis and quality of life. In recent years, advances in biomarker identification and imaging technologies have driven growing research into developing HF risk prediction models for AMI patients post-PCI. However, significant heterogeneity exists across current studies in terms of model construction methods, variable selection, and validation strategies, and the predictive performance and clinical utility of these models have not been systematically evaluated.

**Objective:**

To systematically review published studies on risk prediction models for HF after PCI in AMI patients.

**Design:**

Systematic review and meta-analysis.

**Methods:**

Databases including PubMed, Web of Science, Embase, Cochrane Library, CNKI, VIP, Wanfang Data Knowledge Service Platform, Chinese Journal Full-text Database, and Chinese Biomedical Literature Database were searched from inception to December 31, 2024. Eligibility criteria included: study participants aged ≥ 18 years with AMI post-PCI; studies focused on HF risk prediction model development; and publication in Chinese or English. The Prediction Model Risk of Bias Assessment Tool (PROBAST) was used to evaluate bias risk in model construction and validation, and RevMan 5.4 software was employed for meta-analysis.

**Results:**

A total of 14 studies involving 14 HF risk prediction models for AMI patients post-PCI were included. All studies adopted a retrospective design, with HF incidence ranging from 3.0% to 37.5%. The area under the curve (AUC) for model discriminative ability ranged from 0.847 to 0.966, indicating strong predictive power. Meta-analysis identified 19 effective predictors of post-PCI HF in AMI patients (all *P* < 0.05): Killip classification, age, Gensini score, D-dimer level, N-terminal pro-brain natriuretic peptide (NT-proBNP), neutrophil-to-high-density lipoprotein cholesterol ratio (NHR), neutrophil-to-lymphocyte ratio (NLR), modified shock index (MSI), troponin I, cardiac troponin T, serum creatinine, high-sensitivity C-reactive protein (hs-CRP), ventricular wall motion amplitude, hypertension, time from symptom onset to hospital admission, number of diseased coronary vessels, diabetes mellitus, arrhythmia, and cardiac structural changes. However, all studies were rated as high overall bias risk via PROBAST, primarily due to insufficient sample size, improper handling of continuous variables, and overreliance on univariate analysis for variable selection.

**Conclusion:**

While existing HF risk prediction models for AMI patients post-PCI demonstrate favorable discriminative ability, they suffer from high overall bias risk. Future studies should adhere strictly to PROBAST guidelines, optimize study design, improve methodological quality, and conduct multicenter prospective studies to validate model stability and generalizability. This will facilitate the development of clinically robust, applicable, and evidence-based prediction tools for clinical practice.

**Trial registration:**

CRD42025639512

**Supplementary Information:**

The online version contains supplementary material available at 10.1186/s12872-025-05406-z.

 Acute myocardial infarction (AMI) represents the most severe form of coronary atherosclerotic heart disease, characterized by myocardial ischemia, hypoxia, and necrosis resulting from acute coronary occlusion [1]. As a leading global cause of mortality and disability, AMI imposes a particularly heavy disease burden in China [2]. In recent years, the widespread adoption of percutaneous coronary intervention (PCI), establishment of chest pain center systems, and optimization of pharmacotherapeutic regimens have significantly reduced short-term mortality in AMI patients [3]. Nevertheless, post-PCI patients remain at high risk of heart failure (HF)—a complication that not only compromises long-term prognosis and quality of life but also increases medical resource consumption and economic burden [4].

Epidemiological data underscore the clinical significance of post-PCI HF in AMI patients. For instance, a multicenter cohort study by Li et al. across 50 + hospitals in China reported that over 6.3% of AMI patients required unplanned readmission within 30 days post-PCI, with 16.7% of these readmissions directly attributed to HF [5]. This highlights HF as one of the most common post-PCI complications in AMI patients, with a direct impact on clinical outcomes. Thus, effective prediction and management of post-PCI HF risk have become urgent priorities in cardiovascular medicine.

Current clinical risk assessment tools—such as Killip classification and Gensini score—are widely used to evaluate AMI severity and coronary lesion extent. Killip classification, based on clinical symptoms, effectively reflects cardiac function [6, 7], while Gensini score indirectly quantifies myocardial injury by assessing coronary lesion severity [8, 9]. However, these tools lack specificity for post-PCI HF and may fail to accurately predict risk in distinct patient subgroups. This gap has driven research into developing HF-specific risk prediction models for AMI patients post-PCI.

Recent innovations in biomarker identification and imaging have enabled the integration of multiple predictors (e.g., age, medical history, laboratory indices like NT-proBNP and hs-CRP, and cardiac function parameters such as left ventricular ejection fraction [LVEF]) into prediction models [10]. Despite this progress, heterogeneity in model development, variable selection, and validation limits the generalization of findings. To address this, the present study systematically reviews and meta-analyzes existing models to evaluate their methodological quality, predictive performance, and clinical applicability—ultimately providing evidence for clinical practice and guiding future model optimization.

## Materials and methods

### Protocol and registration

This study was conducted in accordance with the Preferred Reporting Items for Systematic Reviews and Meta-Analyses (PRISMA) guidelines and registered on PROSPERO (Registration No.: CRD42025639512).

### Inclusion and exclusion criteria

Inclusion criteria: (1) The data are incomplete or invalid, and the full text cannot be obtained; (2) Study focus: Development of HF risk prediction models; (3)Study design: Case-control, cohort, or cross-sectional studies; (4)Language: Chinese or English.

Exclusion criteria: (1) Incomplete/invalid data or unavailable full text; (2) Insufficient description of model construction methods; (3) Duplicate publications; (4) No formal prediction model development or missing model data; (5) HF listed as one of multiple outcomes (not the primary focus); (6) Reviews, case reports, conference abstracts, or non-original studies.

### Literature search strategy

Publicly published studies on the construction of risk prediction models for HF after PCI in AMI patients were searched in PubMed, Web of Science, Embase, Cochrane Library, CNKI, VIP, Wanfang Data Knowledge Service Platform, Chinese Journal Full-text Database, and Chinese Biomedical Literature Database. The search period was from the establishment of each database to December 31, 2024. A combination of subject terms and free terms was used for searching, adjusted according to the characteristics of each database, supplemented by reference backtracking.

Chinese search terms: Acute myocardial infarction/acute myocardial infarction, myocardial infarction, percutaneous coronary intervention/PCI, heart failure/heart failure, model, prediction model, risk prediction, predictor, risk stratification, risk score, risk assessment;

English search terms: Myocardial infarction, percutaneous coronary intervention, PCI, heart failure, predict, prediction model, risk prediction, risk assessment, risk evaluation, risk score, risk stratification model, prediction tool, prognostic model, stratification model, post-PCI.

### Literature screening and data extraction

Two independent reviewers screened titles/abstracts against eligibility criteria, followed by full-text review for final inclusion. A data extraction form was pre-designed using the Checklist for Critical Appraisal and Data Extraction for Systematic Reviews of Prediction Modelling Studies (CHARMS) [11]. Discrepancies were resolved via discussion, with a third reviewer consulted if consensus was not reached.

### Quality assessment

The PROBAST tool was used to evaluate bias risk (4 domains, 20 items) and applicability (3 domains) [12]. A study was rated as high overall bias risk if any bias domain was classified as high or unclear; high overall applicability risk was assigned if any applicability domain was high. Two reviewers independently conducted assessments, with discrepancies resolved via discussion or third-party consultation.

### Statistical analysis

RevMan 5.4 was used for meta-analysis of common predictors across models. Heterogeneity was assessed via Q-test and I² statistic: a fixed-effects model was used if *P* > 0.1 and I²<50% (low heterogeneity); otherwise, a random-effects model was applied. Sensitivity analysis was performed for predictors with high heterogeneity. Odds ratios (OR) with 95% confidence intervals (CI) were used as effect measures.

## Results

### Literature screening outcomes

After rigorous screening, 14 studies were included: 12 in Chinese, 2 in English, and 13 published within the past 5 years. All studies adopted a retrospective design, with HF incidence ranging from 3.0% to 37.5%. Baseline characteristics of included studies are summarized in Table 1.

**Table 1:** Basic characteristics of included studies

Author (year)	Country/Region	Study Type	Study Design	Study Subjects	Data Source	HF Incidence (%)
Zhang 2024^[13]^	Hunan, China	D + V	retrospective	First PCI, age > 18	Hospital EHR system + China Chest Pain Center Database	3.0
Song 2024^[14]^	Guangxi, China	D + V	retrospective	PCI, age ≥ 18	Hospital EHR system	A:12.2, B:16.2
Guo 2024^[15]^	Beijing, China	D + V	retrospective	PCI, age ≥ 60	Hospital EHR system	A:33.3, B:37.5
Qian 2024^[16]^	Beijing, China	D + V	retrospective	PCI, age ≥ 60	Hospital EHR system	A:31.5, B:32.1
Chen 2024^[17]^	Zhejiang, China	D	retrospective	Post-PCI patients	Hospital EHR system	16.1
Yang 2024^[18]^	Nanchong, China	D + V	retrospective	First PCI, age ≥ 18	Clinical medical records	28.5
Chen 2024^[19]^	Wuhan, China	D + V	retrospective	PCI, age > 18	Hospital EHR system	A:5.4, B:8.5
Zhou 2022^[20]^	Anhui, China	D + V	retrospective	PCI, age ≥ 18	Hospital EHR system	29.81
Tang 2022^[21]^	Guangdong, China	D	retrospective	PCI, age > 18	Hospital EHR system	31.25
Wang 2022^[22]^	Zhejiang, China	D	retrospective	PCI, age ≥ 18	Hospital EHR system	34.67
Fu 2021^[23]^	Yunnan, China	D + V	retrospective	PCI, age ≥ 18	Hospital EHR system	25.8
Li 2021^[24]^	Guangdong, China	D + V	retrospective	Post-PCI patients	Hospital EHR system	34.16
Sun 2020^[25]^	Gansu, China	D + V	retrospective	Post-PCI patients	Hospital EHR system	28.0
Li 2019^[26]^	Tianjin, China	D + V	retrospective	Post-PCI patients	Hospital EHR system	30.1

A: Modeling cohort; B: Validation cohort; D: Model development-only; D + V: Model development and validation

D + V: Model development and validation, *EHR* Electronic health record

### Model construction details

All 14 studies developed risk prediction models, with total sample sizes ranging from 139 to 715 participants. Key parameters (e.g., variable selection, modeling methods, and sample distribution) are presented in Table 2.

**Table 2:** Construction and predictive performance of HF risk prediction models for AMI patients Post-PCI

Literature	Variable Selection Method	Modeling Method	Sample Size	Model Performance		Validation Method	Missing Data Handling	Continuous Variable Handling
			Modeling/Validation	AUC/CI	Calibration			
Zhang 2024^[13]^	Univariate analysis	Logistic regression	664/-	0.847/-	*H-L test*,*P* = 0.938	Bootstrap internal	-	b
Song 2024^[14]^	Univariate analysis	Lasso regression, logistic regression	473/203	0.87/-	*H-L test*,*P* = 0.3036	Internal	-	b
Guo 2024^[15]^	Univariate analysis	Logistic regression	222/56	0.897/0.567	*H-L test*,*P* = 0.545	Bootstrap internal	-	a
Qian 2024^[16]^	Univariate analysis	Logistic regression	111/28	0.903/0.573	*H-L test*, *P* = 0.532	Bootstrap internal	-	a
Chen 2024^[17]^	Univariate analysis	Logistic regression	193/-	0.893/0.856	Calibration curve	-	-	a
Yang 2024^[18]^	Univariate analysis	Lasso regression, logistic regression	288/-	0.894/0.862	Calibration curve	Bootstrap internal	Delete directly	a
Chen 2024^[19]^	Lasso regression	Logistic regression	503/212	0.966/-	Calibration curve	Bootstrap internal	-	b
Zhou 2022^[20]^	Univariate analysis	Logistic regression	208/-	0.856/-	Calibration curve	Internal	-	a
Tang 2022^[21]^	Univariate analysis	Logistic regression	160/-	-/0.769	Calibration curve	-	-	a
Wang 2022^[22]^	Univariate analysis	Logistic regression	323/-	-/0.821	-	-	-	a
Fu 2021^[23]^	Stepwise backward method	Logistic regression	438/134	-/0.832	*H-L test*, *P* = 0.288	External	-	a
Li 2021^[24]^	Univariate analysis	Full subset regression, random forest	445/-	0.855/0.846	Calibration curve	Internal	-	b
Sun 2020^[25]^	Lasso-logistic regression	Logistic regression	318/-	0.911/0.911	Calibration curve	External	-	a
Li 2019^[26]^	Stepwise backward method	Logistic regression	495/128	-/0.81	Calibration curve	External	-	a

*AUC* Area under the receiver operating characteristic curve,* CI* Concordance index, *H-Ltest* Hosmer-Lemeshow goodness-of-fit test

a: Converted to categorical variables; b: Maintained as continuous variables

### Model predictive performance

Model performance was evaluated by discriminative ability (AUC/C-index) and calibration (H-L test, calibration curves). All 14 studies reported discriminative ability, with AUC ranging from 0.847 to 0.966 (indicating strong ability to distinguish high/low-risk patients). One study omitted calibration data; the remaining 13 used either H-L tests (*P* > 0.05, indicating good calibration) or calibration curves (visually consistent with ideal predictions). Data are presented in Table 2.

### Model presentation

All 14 models were presented as nomograms—an intuitive format favored for clinical use. Key predictors, applicability, and limitations of each model are summarized in Table 3.

**Table 3:** Overview of HF risk prediction models for AMI patients Post-PCI

Literature	No. of Predictors	Model Format	Key Predictors	Applicability & Limitations
Zhang 2024^[13]^	3	Nomogram	Killip classification, renal insufficiency, troponin T	Good applicability; missing potential influential factors.
Song 2024^[14]^	5	Nomogram	Age, troponin, D-dimer, LVEF, Gensini score	Good applicability; lacks external validation.
Guo 2024^[15]^	4	Nomogram	NT-proBNP, NLR, NHR, MSI	Good applicability; small sample size (needs large-scale validation).
Qian 2024^[16]^	4	Nomogram	NT-proBNP, NLR, NHR, MSI	Good applicability; small sample size, limited predictors.
Chen 2024^[17]^	4	Nomogram	Troponin I, left ventricular global longitudinal strain, global wasted work, global work index	Good applicability; single-center design (needs external validation).
Yang 2024^[18]^	7	Nomogram	Anterior descending artery lesion, Gensini score, troponin T, serum creatinine, hs-CRP, total bilirubin, ventricular wall motion amplitude	Good applicability; lacks external validation.
Chen 2024^[19]^	6	Nomogram	Age, hypertension prevalence, CCU admission rate, hospital stay duration, onset-to-admission time, D-dimer	Good applicability; lacks multicenter validation.
Zhou 2022^[20]^	13	Nomogram	Age, diseased vessels, anterior MI, Gensini score, hypertension, diabetes, LVEF, troponin I, CK-MB, NT-proBNP, hs-CRP, revascularization, ventricular wall motion amplitude	Good applicability; single-center, small sample size, long data collection period.
Tang 2022^[21]^	6	Nomogram	Age, Killip classification, blood urea nitrogen, triglycerides, LDL-C, HDL-C	Good applicability; single-center, small sample size.
Wang 2022^[22]^	7	Nomogram	Age, diabetes, cardio-cerebrovascular disease, Gensini grade, hs-CRP, serum creatinine, troponin T	Good applicability; lacks multicenter validation.
Fu 2021^[23]^	7	Nomogram	Age, Gensini score grade, arrhythmia, cardiac structural changes, serum creatinine, troponin T, ventricular wall motion amplitude	Good applicability; single-center (needs prospective validation).
Li 2021^[24]^	6	Nomogram	LVEF, age, white blood cell count, glomerular filtration rate, plasma fibrinogen, heart rate	Good applicability; unproven long-term predictive ability.
Sun 2020^[25]^	8	Nomogram + Simplified Score	Gender, age, 24 h pulmonary rales, onset-to-treatment time, BNP, LVEF, atrial fibrillation, diseased vessels	Easy to use; small sample size (needs further validation for promotion).
Li 2019^[26]^	7	Nomogram	Age, Gensini score, post-infarction arrhythmia, troponin T, serum creatinine, cardiac structural changes, ventricular wall motion amplitude	Good applicability; needs prospective validation.

*LVEF* Left ventricular ejection fraction, *NT-proBNP* N-terminal pro-brain natriuretic peptide, *NLR* Neutrophil-to-lymphocyte ratio, *NHR* Neutrophil-to-high-density lipoprotein cholesterol ratio, *MSI* Modified shock index, *hs-CRP* High-sensitivity C-reactive protein, *CK-MB* Creatine kinase-MB, *LDL-C* Low-density lipoprotein cholesterol, *HDL-C *High-density lipoprotein cholesterol,* CCU* Coronary care unit

### Bias risk and practicality assessment

The PROBAST tool was used to evaluate the risk of bias and applicability of the 14 included studies, and the evaluation results were carefully reviewed to ensure accuracy. In the study population domain, all studies were rated as high risk of bias due to their retrospective design; in the predictor and outcome domains, all were rated as low risk of bias; in the statistical analysis domain, all were rated as high risk of bias. In the applicability evaluation domain, all 14 studies were rated as low risk in all domains and overall evaluation. See Table 4 for details.

To further evaluate the clinical practical value of the included models, this study developed a practicality grading standard based on four core dimensions: completeness of external validation, adequacy of sample size/number of events, accessibility of predictive variables, and convenience of deployment, and graded the 14 models. The specific characteristics and grading results are shown in Table 5.

**Table 4:** Risk of bias and applicability evaluation of included studies

Literature	Risk of Bias				Applicability			Overall	
	Study Population	Predictors	Outcome	Analysis	Study Population	Predictors	Outcome	Bias	Applicability
Zhang 2024^[13]^	+	-	-	+	+	+	+	-	+
Song 2024^[14]^	+	-	-	+	+	+	+	-	+
Guo 2024^[15]^	+	-	-	+	+	+	+	-	+
Qian 2024^[16]^	+	-	-	+	+	+	+	-	+
Chen 2024^[17]^	+	-	-	+	+	+	+	-	+
Yang 2024^[18]^	+	-	-	+	+	+	+	-	+
Chen 2024^[19]^	+	-	-	+	+	+	+	-	+
Zhou 2022^[20]^	+	-	-	+	+	+	+	-	+
Tang 2022^[21]^	+	-	-	+	+	+	+	-	+
Wang 2022^[22]^	+	-	-	+	+	+	+	-	+
Fu 2021^[23]^	+	-	-	+	+	+	+	-	+
Li 2021^[24]^	+	-	-	+	+	+	+	-	+
Sun 2020^[25]^	+	-	-	+	+	+	+	-	+
Li 2019^[26]^	+	-	-	+	+	+	+	-	+

Note: +: Low risk of bias, high applicability; -: High risk of bias, low applicability

**Table 5:** Grading evaluation table of model practicality

Model (Author/Year)	External Validation	Sample Size/Number of Events (Modeling Cohort)	Accessibility of Predictive Variables (Routine Test = 1, Special Test = 0)	Deployment Method	Practicality Grade
Zhang 2024^[13]^	None	664/20	3/3 (Killip Classification, Renal Insufficiency, Troponin T)	Nomogram	Medium
Song 2024^[14]^	None	473/58	5/5 (Age, Troponin, D-dimer, LVEF, Gensini Score)	Nomogram	Medium
Guo 2024^[15]^	None	222/74	4/4 (NT-proBNP, NLR, NHR, MSI)	Nomogram	Low
Qian 2024^[16]^	None	111/35	4/4 (NT-proBNP, NLR, NHR, MSI)	Nomogram	Low
Chen 2024^[17]^	None	193/31	3/4 (Troponin I, GLS requiring special ultrasonic analysis)	Nomogram	Low
Yang 2024^[18]^	None	288/82	6/7 (Total Bilirubin requiring biochemical testing)	Nomogram	Medium
Chen 2024^[19]^	None	503/45	6/6 (CCU Admission History, Length of Hospital Stay)	Nomogram	Medium
Zhou 2022^[20]^	None	208/62	12/13 (Revascularization History)	Nomogram	Medium
Tang 2022^[21]^	None	160/50	6/6 (Triglyceride, LDL-C)	Nomogram	Low
Wang 2022^[22]^	None	323/112	7/7 (Cardio-Cerebrovascular Disease History, Gensini Classification)	Nomogram	Medium
Fu 2021^[23]^	Yes	438/113	7/7 (Arrhythmia, Cardiac Structural Changes)	Nomogram	High
Li 2021^[24]^	None	445/152	6/6 (White Blood Cell Count, Glomerular Filtration Rate, Fibrinogen)	Nomogram	Medium
Sun 2020^[25]^	Yes	318/89	8/8 (24-hour Pulmonary Rales on Admission, Atrial Fibrillation History)	Nomogram + Simplified Scoring Table	High
Li 2019^[26]^	Yes	495/149	7/7 (Post-infarction Arrhythmia, Cardiac Structural Changes)	Nomogram	High

Grading Criteria:

High: Meets the criteria of "having external validation + sample size >400/number of events >100 + all predictive variables are routine tests + convenient deployment method"

Medium: Meets the criteria of "no external validation but with internal validation + sample size 200–400/number of events 50–100 + no less than 80% of the predictive variables are routine tests + nomogram only"

Low: Meets the criteria of "no validation + sample size < 200/number of events < 50 + no less than 20% of the predictive variables are special tests + nomogram only"

### Meta-analysis of predictors

Further meta-analysis of common predictors in each model showed that Killip classification, age, Gensini score, D-dimer level, NT-proBNP, NHR, NLR, MSI, troponin I, cardiac troponin T, serum creatinine, hs-CRP, ventricular wall motion amplitude, hypertension, time from onset to treatment, number of diseased vessels, diabetes mellitus, arrhythmia, and cardiac structural changes were effective predictors of HF after PCI in AMI patients (*P* < 0.05). Sensitivity analysis was performed for predictors with high heterogeneity, and the combined statistical results showed no significant changes, indicating that the meta-analysis results were relatively stable; see Table 6 for details.

**Table 6:** Meta-Analysis results of HF predictors in AMI patients Post-PCI

Predictor	No. of Studies	Heterogeneity		Effect Model	Meta Analysis	
		I^2^(%)	*P* Value		OR(95%CI)	*P* Value
Killip classification	2	0	0.95	fixed	3.06(1.68,5.56)	< 0.001
Age	9	92	< 0.001	random	1.77(1.29,2.41)	< 0.001
Gensini score	6	91	< 0.001	random	7.06(1.73,28.75)	0.006
LVEF	4	90	< 0.001	random	1.02(0.75,1.39)	0.910
D-dimer level	2	0	0.70	fixed	2.64(1.56,4.47)	< 0.001
NT-proBNP	3	0	0.89	fixed	10.49(5.26,20.91)	< 0.001
NHR	2	0	0.94	fixed	9.25(3.05,28.01)	< 0.001
NLR	2	0	1.00	fixed	10.83(3.51,33.43)	< 0.001
MSI	2	0	0.99	fixed	8.27(2.67,25.57)	< 0.001
Troponin I	2	93	< 0.001	fixed	1.36(1.15,1.60)	< 0.001
Cardiac troponin T	4	71	0.01	random	1.42(1.01,2.00)	0.04
Serum creatinine	4	39	0.18	random	6.67(2.42,18.42)	< 0.001
hs-CRP	3	50	0.13	random	10.22(2.32,45.00)	0.002
Ventricular wall motion amplitude	4	51	0.10	random	5.37(1.90,15.15)	0.002
Hypertension	2	38	0.20	fixed	4.27(2.11,8.65)	< 0.001
Onset-to-treatment time	2	51	0.15	fixed	2.24(1.43,3.52)	< 0.001
Diseased vessels	2	70	0.07	fixed	5.46(2.63,11.36)	< 0.001
Diabetes mellitus	2	0	0.51	fixed	4.50(2.28,8.91)	< 0.001
Arrhythmia	2	0	0.81	fixed	2.05(1.33,3.16)	0.001
Cardiac structural changes	2	0	0.95	fixed	2.43(1.39,4.23)	0.002

*LVEF* Left ventricular ejection fraction, *NT-proBNP* N-terminal pro-brain natriuretic peptide, *NLR* Neutrophil-to-lymphocyte ratio, *NHR* Neutrophil-to-high-density lipoprotein cholesterol ratio, *MSI* Modified shock index, *hs-CRP* High-sensitivity C-reactive protein

## Discussion

### Comprehensive evaluation of model predictive performance, risk of bias, and clinical practicality

This study systematically reviewed the latest research on risk prediction models for HF after PCI in AMI patients. The results showed that existing models exhibit promising predictive potential in terms of discriminative ability (AUC: 0.847~0.966), indicating their good ability to identify high-risk patients. However, the evaluation of model quality and transformation value needs to go beyond simple discriminative indicators. The PROBAST tool assessment revealed a key contradiction: despite high predictive performance, all included studies had high risk of bias in the "study population" (all retrospective designs) and "statistical analysis" (such as insufficient sample size, improper handling of continuous variables, and single variable selection method), which seriously restricts the reliability and extrapolatability of model results.

Further comprehensive analysis combined with model practicality grading (Table 5) clearly outlined the three-tier structure of the current research ecosystem:

Top tier (High practicality models, 3 studies): Common features include completion of external validation, relatively sufficient sample size/number of events, all predictive variables being routine clinical tests, and convenient deployment. These set a preliminary benchmark for "usable" and even "easy-to-use" models in the future.

Middle tier (Medium practicality models, 7 studies): Constituting the main body of the research, most conducted internal validation and adopted nomogram presentation. However, due to the lack of external validation, limited sample size, or poor accessibility of some variables, their clinical promotion value is uncertain.

Bottom tier (Low practicality models, 4 studies): Limited by small sample size, lack of validation, and poor accessibility of variables, they are currently mainly confined to methodological exploration.

In summary, this study identified the core challenge in the current field as the"imbalance among predictive performance, risk of bias, and clinical practicality". The grading results in Table 5 clearly show that improving predictive performance alone cannot naturally realize clinical transformation of models. High risk of bias fundamentally undermines the scientific foundation of prediction models, while low practicality directly hinders their integration into clinical workflows.

Therefore, future studies must establish a trinity model construction goal of "high predictive performance, low risk of bias, and high clinical practicality". To achieve this goal, this study proposes a two-dimensional optimization path: first, in terms of study design and methodology, strictly follow the PROBAST and TRIPOD guidelines, and strive to reduce the risk of bias from"high" to "low". Specific measures include prioritizing prospective designs, standardizing the processing of continuous variables and variable selection processes, properly handling missing data, and expanding sample size. Second, in terms of model transformation, actively carry out multicenter external validation, ensure the routine accessibility of predictors, and develop more convenient deployment tools (such as online calculators integrated into electronic medical record systems). Through such systematic efforts that balance methodological rigor and clinical transformation needs, we can promote HF prediction models after AMI-PCI from"high performance on paper" to "high value at the bedside", and ultimately provide effective decision support for improving patient prognosis.

### Effective predictors of heart failure after PCI in patients with acute myocardial infarction

Through meta-analysis of common predictors in the included models, this study identified multiple predictors significantly associated with the occurrence of HF after PCI in AMI patients. These predictors include Killip classification, age, Gensini score, D-dimer level, NT-proBNP, NHR, NLR, MSI, troponin I, cardiac troponin T, serum creatinine, hs-CRP, ventricular wall motion amplitude, hypertension, time from onset to treatment, number of diseased vessels, diabetes mellitus, arrhythmia, and cardiac structural changes.

#### Killip classification and Gensini score: important clinical assessment indicators

As traditional clinical assessment tools, Killip classification and Gensini score are widely used to evaluate the severity of illness in AMI patients. Killip classification is based on patients’ clinical symptoms, and studies have shown [6, 7] that it plays an important role in predicting postoperative HF. A higher Killip classification usually reflects more severe cardiac insufficiency, so Killip classification can effectively predict the risk of HF. In addition, Gensini score indirectly reflects the degree of myocardial injury by assessing the severity of coronary artery lesions. A higher Gensini score indicates more severe coronary artery lesions in patients, which usually leads to greater myocardial damage and a higher risk of HF [27–30]. Therefore, Gensini score is also of great significance in predicting HF after PCI.

#### Diagnostic value of biomarkers

In recent years, biomarkers have gradually become important tools for predicting HF in AMI patients. NT-proBNP, as a sensitive marker of HF, has been extensively studied and proven to have high sensitivity and specificity in predicting HF. The level of NT-proBNP is closely related to the severity of congestive heart failure, and an increase in NT-proBNP after surgery usually indicates the occurrence of HF [31–34]. Therefore, NT-proBNP monitoring after PCI has important clinical significance. D-dimer and hs-CRP are biomarkers reflecting inflammatory response and coagulation status, and their increased levels are also closely related to the occurrence of HF. An increase in D-dimer suggests that the patient may have thrombosis, which further aggravates HF symptoms [35], while hs-CRP reflects the chronic inflammatory response in the body, and these factors may increase the risk of HF [36].

#### Impact of clinical characteristics on heart failure

Patients’ underlying diseases, such as age, hypertension, and diabetes mellitus, play an important role in the occurrence of postoperative HF. With the increase of age, the function of the cardiovascular system gradually declines, and the myocardial repair ability weakens, leading to a higher risk of HF in elderly patients [37]. Hypertensive patients are often accompanied by left ventricular hypertrophy, myocardial injury, and changes in cardiac structure. These pathological changes make hypertensive patients more prone to HF after PCI [38]. Diabetes mellitus further increases the burden on the heart through mechanisms such as glucose metabolism disorders and atherosclerosis, leading to an increased risk of HF in patients [39]. Therefore, these patients have a higher cardiovascular risk, and special attention should be paid to the prediction and management of postoperative HF.

#### Role of inflammatory response

NHR (neutrophil-to-high-density lipoprotein cholesterol ratio) and NLR (neutrophil-to-lymphocyte ratio) are inflammatory response markers that have attracted attention in recent years. Studies have shown that their increase is closely related to the risk of HF after acute myocardial infarction [40–43]. An increase in neutrophils usually indicates a strong inflammatory response in the body, which can aggravate myocardial injury and further induce HF [44]. An increase in NHR and NLR may indicate a strong inflammatory response in patients, increasing the risk of postoperative HF. Therefore, these indicators have certain early warning value as inflammatory assessment tools in clinical practice.

#### Changes in cardiac function and structure

Troponin I and troponin T are common markers of myocardial injury. An increase in these markers after surgery usually indicates further myocardial injury. The increase of troponin can reflect the existence of myocardial injury in the early stage of acute myocardial infarction and is closely related to the occurrence of HF [45, 46]. In addition, an increase in serum creatinine often reflects renal insufficiency. There is an interaction between renal and cardiac functions, and renal failure may aggravate HF symptoms. Therefore, monitoring serum creatinine levels is of great significance for predicting the risk of HF after PCI [47, 48]. Changes in ventricular wall motion amplitude can also be used as an indicator of cardiac function injury. A decrease in ventricular wall motion amplitude shown by cardiac ultrasound often means impaired myocardial systolic function, which may indicate the occurrence of HF [49–51].

Among them, Killip classification and Gensini score are important indicators reflecting patients’ cardiac function and the severity of coronary artery lesions, which are closely related to the occurrence of HF. Patient-related factors such as age, hypertension, and diabetes mellitus are also important predictors. These factors may increase the risk of HF by affecting vascular function and myocardial repair ability. In addition, biomarkers such as NT-proBNP, D-dimer, and hs-CRP show high sensitivity and specificity in predicting HF, suggesting that these indicators have important application value in clinical practice.

### Future research directions and implications

Although existing models show good performance in predicting the risk of HF after PCI in AMI patients, they still have limitations and need to be optimized in the following directions.

#### Research progress and application of artificial intelligence in China

Some studies have used machine learning algorithms such as Lasso regression and random forest to construct models, and their predictive performance is better than traditional logistic regression. The latest research published by Chinese scholars in 2025 further focuses on model interpretability, coverage of subpopulations, and clinical transformation and implementation, providing more solid local evidence for the application of AI technology in predicting HF after AMI-PCI:

A single-center retrospective study by Guo et al. (*n* = 1574 AMI patients) first applied the TabNet deep learning model to predict the severity of HF (Killip classification) after surgery, and compared the performance of random forest (RF), XGBoost, and multi-layer perceptron (MLP) models. The results showed that the TabNet model performed the best: the AUC for Killip four-classification prediction was 0.827, and for two-classification prediction was 0.831, which was significantly better than traditional machine learning models. The study identified GRACE score, TIMI score, NT-proBNP, LVEF, and creatinine clearance rate (CCR) as key predictors through the SHAP method. Among them, increased NT-proBNP and decreased LVEF were significantly associated with higher Killip classification, which was completely consistent with the conclusion of this meta-analysis that “NT-proBNP and LVEF are effective predictors”; at the same time, a directly clinically applicable web platform was developed and made public (https://prediction-killip-gby.streamlit.app/), realizing the closed loop of “model construction - interpretation - clinical transformation” [52].

A single-center prospective study by Wang et al. focused on the special population of premature myocardial infarction (PMI, males ≤ 50 years old, females ≤ 55 years old) and constructed a prediction model for in-hospital heart failure with preserved ejection fraction (HFpEF). The study included 840 PMI patients (268 developed HFpEF, with an incidence of 31.90), and compared 5 algorithms including Lasso-Logistic, XGBoost, and random forest. The results showed that the XGBoost model performed the best, significantly better than other models. The model finally included 10 key predictors, including BNP >100 pg/ml, SYNTAX score >14.5, age, and monocyte-to-lymphocyte ratio (MLR) >0.3. Among them, increased BNP and complex coronary lesions were strongly associated with an increased risk of HFpEF, which was highly consistent with the conclusion of this meta-analysis that “biomarkers and the degree of coronary artery lesions are core predictors”; at the same time, a visual prediction system was developed (https://hfpefpmi.shinyapps.io/apppredict/), which supports clinicians to quickly obtain the probability of HFpEF and SHAP explanation diagrams after inputting patients’ clinical indicators, filling the gap in predicting specific HF subtypes in young AMI patients [53].

A single-center retrospective study by Lin et al. constructed a prediction model for the 3-year risk of HF after PCI in AMI patients, including 1220 patients (244 developed HF, with an incidence of 20%). Six key factors (LDH, CK-MB, hsCRP, NT-proBNP, LVEF, and left ventricular end-systolic diameter (LVDs)) were selected through LASSO regression, and the performance of XGBoost, random forest, SVM, and logistic regression models was compared. The results showed that the XGBoost model performed the best. SHAP analysis identified LVEF, LVDs, and LDH as the three most important predictors. Among them, the risk of HF was significantly increased when LVEF < 50%, LVDs >4.0 cm, and LDH >650 U/L, which was consistent with the conclusion of this meta-analysis that “LVEF, myocardial injury markers, and cardiac structural changes are effective predictors”; at the same time, individual-level prediction interpretation was realized through SHAP waterfall diagrams and force-directed diagrams, clearly showing the contribution of each factor to the patient’s risk, and providing an intuitive tool for clinical personalized assessment [54].

The above studies in 2025 show three core advantages: first, stronger algorithm adaptability, including deep structured models such as TabNet (suitable for multi-classification tasks) and mature integrated algorithms such as XGBoost (suitable for subtype-specific and long-term risk prediction), which can meet the needs of different clinical scenarios; second, comprehensive enhancement of model interpretability, all using the SHAP method to reveal the prediction logic, and the core predictors are highly consistent with the results of this meta-analysis, further verifying clinical generality; third, prominent clinical transformation and implementation, two studies developed publicly accessible visual platforms, which can quickly obtain prediction results without complex operations, reducing the threshold for clinical application.

At the same time, limitations should be viewed objectively: all three studies were single-center designs, without multicenter external validation, and the extrapolatability of the models in different medical environments needs to be verified; the predictors are still mainly structured clinical indicators and biomarkers, without fully integrating unstructured data such as radiomics and genomic data, which may miss potential prediction information; there is no targeted prediction tool for complex populations with multiple underlying diseases. Future studies can promote multicenter prospective validation of models on the existing basis, explore the integration mode of “structured data + multi-modal data”, and develop special models focusing on more complex subpopulations to further improve the generality, accuracy, and clinical transformation value of AI models.

#### Optimization of study design and methodology

All 14 existing studies were conducted by Chinese scholars, and the model presentation method was nomogram. Although nomogram is an intuitive risk prediction tool easily accepted by clinicians, its generality and extrapolatability still need to be verified. Current studies are mostly single-center retrospective designs, lacking external validation across regions and ethnic groups, which limits the clinical application scope of the models. In the future, it is necessary to strictly follow the PROBAST tool and TRIPOD statement to optimize study design: prioritize multicenter, prospective cohort studies to expand sample size and cover different regional medical environments; standardize the processing of continuous variables (verify the linear relationship before deciding whether to classify), missing data (adopt multiple imputation instead of direct deletion), and variable selection (avoid relying solely on univariate analysis); explore the differences in HF risk among different regions and medical resource conditions for the Chinese population, and construct more generalizable prediction models.

#### Model presentation and clinical transformation

Existing models are mainly nomograms. In the future, the integration mode of “AI algorithm + nomogram” can be explored to retain the intuitiveness of nomograms while combining the advantages of AI in processing high-dimensional data to improve prediction accuracy; in addition, models can be combined with dynamic risk scoring systems and hospital electronic medical record systems to develop convenient clinical decision support tools, enhancing the practicality and user experience of models; at the same time, international cooperation should be strengthened to include patient data from different ethnic groups and regions, verify the applicability of models in global populations, and promote the international application of models.

### Limitations of the study

This study has the following limitations, which should be carefully considered when interpreting the results and conclusions:

Limitations in literature search and representativeness of included populations: This study only searched Chinese and English databases and published literature, not including gray literature and studies in other languages, which may lead to potential publication bias and literature omission. In addition, all 14 included studies were from China, lacking verification in populations of different ethnic groups, regions, and medical systems. Therefore, the predictors integrated by the meta-analysis of this study and the model practicality evaluation results have limited extrapolatability worldwide.

Insufficient breadth and depth of model validation: Among the included models, only 3 (21.4%) were rated as “high practicality” and completed external validation, and most of these validations were conducted in single centers or small scopes, lacking extensive, prospective multicenter validation across different geographical regions and medical institutions at all levels. The remaining 11 models (78.6%) did not undergo external validation, and their predictive performance and stability are highly dependent on internal validation results, making the existing evidence system’s support for the performance of models in real-world clinical environments still weak.

Methodological quality and risk of bias: According to the PROBAST tool assessment, all included studies had high risk of bias, mainly due to the inherent limitations of retrospective study design and common methodological deficiencies in some studies, such as insufficient sample size and number of outcome events (failing to meet the empirical criterion of “at least 10–20 events per predictive variable” in prediction model development), over-reliance on univariate analysis for initial variable screening, artificial classification of continuous variables without pre-testing their linear relationship with outcomes, and unclear or improper handling of missing data. These factors may affect the accuracy and reliability of model parameter estimation.

Imperfection of practicality evaluation criteria: This study independently constructed a model practicality grading standard based on four dimensions: external validation, sample size, variable accessibility, and deployment method. Although the standard strives to be objective, it has not been cross-validated with existing international model practicality or implementability assessment tools, and its assessment validity needs further confirmation in future studies. At the same time, the current assessment mainly focuses on the “theoretical practicality” at the model construction and reporting level, and has not yet evaluated the “real-world impact” of models on diagnosis and treatment decisions and patient prognosis after clinical application. The evaluation dimensions need to be expanded in the future.

Limitations of predictors and model functions: The effective predictors identified through meta-analysis are mainly traditional clinical characteristics, routine laboratory biomarkers, and basic imaging parameters, lacking exploration and integration of new multi-omics indicators such as radiomics and genetic markers. In addition, all included models aim to predict the binary outcome of “whether HF occurs”, and cannot perform more refined risk stratification prediction on the severity, subtype, or occurrence time of HF, thus limiting their potential value in individualized precise management to a certain extent.

Timeliness gap between research data and clinical practice: The original medical record data of some included studies can be traced back to earlier years (as early as before 2019). In recent years, the acute phase management strategies, PCI technology, and guideline-directed pharmacotherapy for acute myocardial infarction have been continuously optimized and updated. These changes in diagnosis and treatment models may potentially alter the incidence of HF and the strength of its association with traditional predictors. Therefore, the applicability and predictive performance of some models constructed based on historical data may need to be re-evaluated and updated in the context of current best clinical practice.

## Conclusion

Through systematic review and meta-analysis, this study comprehensively evaluated the existing risk prediction models for HF after PCI in AMI patients. The results showed that existing models exhibit relatively ideal predictive potential in terms of discriminative ability, and meta-analysis also identified multiple predictors significantly associated with the occurrence of HF, providing valuable references for clinical identification of high-risk populations. However, the PROBAST tool assessment indicated that all included studies had a high risk of bias, mainly due to methodological limitations such as retrospective design, insufficient sample size, irregular variable handling, and inadequate validation. In addition, model practicality grading showed that only a few models have good clinical transformation potential.

In summary, although existing models show certain predictive performance, their methodological quality and clinical extrapolatability still need to be improved. Future studies should strictly follow the relevant specifications for prediction models, and strive to systematically reduce the risk of bias through prospective design, multicenter cooperation, standardized statistical methods, expanded sample size and number of events, and strengthened internal and external validation. At the same time, attention should be paid to the clinical accessibility of predictors and the convenience of model deployment, promoting the transformation of models from methodological construction to real-world application, so as to ultimately provide clinical practice with scientific, robust, and practical risk assessment tools to assist in the prevention, treatment, and individualized management of HF after PCI in AMI patients.

## Supplementary Information

Supplementary Material 1.

Supplementary Material 2.

Supplementary Material 3.

Supplementary Material 4.

Supplementary Material 5.

## Data Availability

Data sharing is not applicable to this article as no datasets were generated or analysed during the current study.

## Abbreviations

AMI: Acute myocardial infarction
PCI: Percutaneous coronary intervention
AUC: Area under the curve
LVEF: Left ventricular ejection fraction
NT-proBNP: N-terminal pro-brain natriuretic peptide
NLR: Neutrophil to lymphocyte ratio
NHR: Ratio of neutrophil to high density lipoprotein cholesterol
MSI: Modified shock index
Hs-CRP: High-sensitivity c-reactive protein
HF: Heart failure
CI: Concordance index
H-L test: Hosmer-Lemeshow goodness-of-fit test
OR: Odds ratios
CK-MB: Creatine kinase-MB
LDL-C: Low-density lipoprotein cholesterol
HDL-C: High-density lipoprotein cholesterol
CCU: Coronary care unit

## References

[1] Reed GW, Rossi JE, Cannon CP, et al. Acute myocardial infarction[J]. Lancet. 2017;389(10065):197–210. 27502078 10.1016/S0140-6736(16)30677-8

[2] Summary of the Report on Cardiovascular Health. Diseases in China 2022[J]. Chin Circ J. 2023;38(6):583–612.

[3] Krämer C, Meisinger C, Kirchberger I, et al. Epidemiological trends in mortality, event rates and case fatality of acute myocardial infarction from 2004 to 2015: results from the KORA MI registry[J]. Ann Med. 2021;53(1):2142–52. 34779325 10.1080/07853890.2021.2002926 PMC8604473

[4] Hao G, Wang X, Chen Z, et al. Prevalence of heart failure and left ventricular dysfunction in china: the China hypertension Survey, 2012–2015[J]. Eur J Heart Fail. 2019;21(11):1329–37. 31746111 10.1002/ejhf.1629

[5] Li J, Dharmarajan K, Bai X, et al. Thirty-day hospital readmission after acute myocardial infarction in China[J]. Circ Cardiovasc Qual Outcomes. 2019;12(5):e005628. 31092023 10.1161/CIRCOUTCOMES.119.005628

[6] Granger CB, Goldberg RJ, Dabbous O, et al. Predictors of hospital mortality in the global registry of acute coronary events[J]. Arch Intern Med. 2003;163:2345–53. 14581255 10.1001/archinte.163.19.2345

[7] Chen DS, Luan XT, Yang JG, et al. Clinical characteristics, treatment and prognosis of Chinese AMI patients with different Killip classifications[J]. Chin Circ J. 2016;31(9):849–53.

[8] Gensini GG. A more meaningful scoring system for determining the severity of coronary heart disease[J]. Am J Cardiol. 1983;51(3):606. 6823874 10.1016/s0002-9149(83)80105-2

[9] He Q, Zhang P, Li Y, et al. The application of Gensini score and IL-1ra in assessing the condition and prognosis of patients with coronary artery disease[J]. Am J Transl Res. 2021;13(9):10421–7. 34650711 PMC8506997

[10] Yang JX, Peng Y, Lin YY, et al. Risk factors and research progress of risk prediction models for heart failure in AMI patients after PCI[J]. Sichuan Med J. 2024;45(3):305–8.

[11] Moons KG, de Groot JA, Bouwmeester W, et al. Critical appraisal and data extraction for systematic reviews of prediction modelling studies: the CHARMS checklist[J]. PLoS Med. 2014;11(10):e1001744. 25314315 10.1371/journal.pmed.1001744 PMC4196729

[12] Moons KGM, Wolff RF, Riley RD, et al. PROBAST: A tool to assess risk of bias and applicability of prediction model studies: explanation and elaboration[J]. Ann Intern Med. 2019;170(1):W1–33. 30596876 10.7326/M18-1377

[13] Zhang L, Liu Z, Zhu Y, et al. A diagnostic prediction model for the early detection of heart failure following primary PCI in patients with STEMI[J]. Am J Cardiovasc Dis. 2024;14(4):208–19. 39309114 10.62347/SHPZ1673 PMC11410788

[14] Song H, Qin WB, Yang FF, et al. Risk analysis and risk prediction of in-hospital heart failure in AMI patients after emergency PCI[J]. BMC Cardiovasc Disord. 2024;24(1):673. 39587472 10.1186/s12872-024-04357-1 PMC11590280

[15] Guo M, Gao Y. Risk factors analysis and nomogram prediction model construction of in-hospital heart failure in elderly patients with STEMI[J]. J Xinjiang Med Univ. 2024;47(11):1496–500.

[16] Qian YY, Tian HT, Liu B, et al. Construction of a nomogram to predict in-hospital heart failure in elderly patients with STEMI[J]. Chin J Lab Diagn. 2024;28(10):1135–40.

[17] Chen JW, Wang XD, Weng WC, et al. Prediction model and efficacy evaluation of heart failure in AMI patients after PCI[J]. Zhejiang Med J. 2024;46(18):1938–43.

[18] Yang JX. Risk prediction model for heart failure after primary PCI in acute ST segment elevated myocardial infarction[D]. North Sichuan Med Coll. 2024. 10.27755/d.cnki.gcbyx.2024.000202.

[19] Chen H. A diagnostic and predictive model of heart failure risk after PCI for acute ST-segment elevation myocardial infarction in Wuhan: a case-control study[D]. Jianghan Univ; 2024. 10.27800/d.cnki.gjhdx.2024.000427.

[20] Zhou CL, Jin XY, Zhao J, et al. Study on nomogram prediction model for heart failure after PCI in AMI patients[J]. Chin J Cardiovasc Rehabil Med. 2022;31(5):586–90.

[21] Tang YP, Li SJ, Su ZD. Analysis of influencing factors and construction of prediction model for heart failure in AMI patients[J]. South China J Cardiovasc Dis. 2022;28(5):438–43.

[22] Wang B, Hu GH, Chen LM, et al. Analysis of influencing factors and establishment of prediction model for postoperative heart failure in AMI patients[J]. Mod Pract Med. 2022;34(8):1002–5.

[23] Fu F, Peng YH, Xu ZY, et al. Study on risk prediction model for heart failure in AMI patients[J]. Chin J Cardiovasc Med. 2021;26(6):525–30.

[24] Li X. Study of risks factors and predictive models of heart failure after ST-segment elevation myocardial infarction[D]. Guangdong Med Univ; 2021.

[25] Sun RM. Risk Prediction Models and Risk Factors for Early Heart Failure after Acute Myocardial Infarction[D]. Lanzhou Univ. 2020. 10.27204/d.cnki.glzhu.2020.003235.

[26] Li YY, Zhang YF, Xu Y, et al. Establishment and evaluation of a risk prediction model for heart failure after PCI in AMI patients[J]. J Clin Cardiol. 2019;35(10):916–22.

[27] Wang K, Zheng Y, Wu T, et al. Predictive value of Gensini score in the Long-Term outcomes of CAD patients who underwent PCI[J]. Front Cardiovasc Med. 2022;8:778615. 35141291 10.3389/fcvm.2021.778615 PMC8818732

[28] Yokokawa T, Yoshihisa A, Kiko T, et al. Residual Gensini score is associated with Long-Term cardiac mortality in HF patients after PCI[J]. Circ Rep. 2020;2(2):89–94. 33693213 10.1253/circrep.CR-19-0121 PMC7929761

[29] Li XT, Huang CL, Chen XJ, et al. Predictive value of Gensini score in the prognosis of STEMI patients after complete revascularization[J]. Chin J Cardiovasc Med. 2023;28(5):411–6.

[30] Liang JZ, Liao SY. Predictive value of Gensini score combined with NT-proBNP for heart failure after PCI in AMI patients[J]. Chin J Cardiovasc Res. 2022;20(6):564–70.

[31] Nowak RM, Christenson RH, Jacobsen G, et al. Performance of novel High-Sensitivity cardiac troponin I assays for 0/1-Hour and 0/2- to 3-Hour evaluations for AMI: results from the HIGH-US Study[J]. Ann Emerg Med. 2020;76(1):1–13. 32046869 10.1016/j.annemergmed.2019.12.008

[32] Du TT, Miao YY, Liu X, et al. Predictive value of plasma BNP for readmission in elderly patients with acute exacerbation of chronic heart failure[J]. Chin J Geriatr. 2018;37(11):1204.

[33] Palà E, Pagola J, Juega J, et al. B-type natriuretic peptide over NT-proBNP to predict incident atrial fibrillation after cryptogenic stroke[J]. Eur J Neurol. 2021;28(2):540–7. 33043545 10.1111/ene.14579

[34] Aşkın L, Tanrıverdi O. The benefits of Sacubitril-Valsartan in low ejection fraction heart Failure[J]. Abant Med J. 2022;11(3):337–336.

[35] Chen RZ. Residual risk and pharmacotherapy after PCI in AMI patients[D]. Peking Union Med Coll. 2023. 10.27648/d.cnki.gzxhu.2023.000412.

[36] Wang XY. Correlation study of GDF-15, NT-proBNP, hs-CRP and the diagnosis of chronic heart failure[D]. Dali Univ. 2022. 10.27811/d.cnki.gdixy.2022.000287.

[37] Ezekowitz JA, Kaul P, Bakal JA, et al. Declining in-hospital mortality and increasing heart failure incidence in elderly patients with first myocardial infarction[J]. J Am Coll Cardiol. 2009;53(1):13–20. 19118718 10.1016/j.jacc.2008.08.067

[38] He ZX, Tian J, Han GF, et al. Impact of in-hospital blood pressure level and changes on all-cause mortality in chronic heart failure patients[J]. Chin J Hypertens. 2024;32(1):57–64.

[39] Dunlay SM, Givertz MM, Aguilar D, et al. Type 2 diabetes mellitus and heart failure: A scientific statement from the AHA and HFSA[J]. J Card Fail. 2019;25(8):584–619. 31174952 10.1016/j.cardfail.2019.05.007

[40] Guo JC. The Correlation of Neutrophil to High Density Lipoprotein Cholesterol Ratio for In-hospital Major Adverse Cardiac Events and Severe Coronary Artery Stenosis in Patients with ST-segment Elevation Acute Myocardial Infarction Following Percutaneous Coronary Intervention[D]. Anhui Med Univ. 2024. 10.26921/d.cnki.ganyu.2024.001264.

[41] Chen J, Liu A, Zhang D, et al. Neutrophil to high-density lipoprotein cholesterol ratio predicts left ventricular remodeling and MACE after PCI in STEMI patients[J]. Front Cardiovasc Med. 2025;12:1497255. 40248256 10.3389/fcvm.2025.1497255 PMC12003286

[42] Rezig AOM, Morsy MI, Caiazzo E, et al. Neutrophil-to-lymphocyte ratio: link to congestion, inflammation, and mortality in outpatients with heart failure[J]. ESC Heart Fail. 2025. 10.1002/ehf2.15678. 40024803 10.1002/ehf2.15240 PMC12055385

[43] Powrózek T, Skwarek-Dziekanowska A, Sobieszek G, et al. Correlation between NLR, PLR, CRP/Albumin ratio and clinical picture of elderly chronic heart failure Patients[J]. J Clin Med. 2024;13(2):433. 38256568 10.3390/jcm13020433 PMC10817038

[44] Frangogiannis NG. The inflammatory response in myocardial injury, repair, and remodelling[J]. Nat Rev Cardiol. 2014;11(5):255–65. 24663091 10.1038/nrcardio.2014.28 PMC4407144

[45] Zhang YT. Clinical value of high sensitive cardiac troponin T, high sensitive C reactive protein, growth differentiation factor-15 and N-terminal pro-brain natriuretic peptidein in patients with heart failure[D]. Shandong Univ. 2018.

[46] Xu CL, Wang QG. Relationship between serum troponin I level and myocardial injury in elderly heart failure patients and ROC analysis[J]. Chin J Gerontol. 2015;35(11):2979–80.

[47] Ronco C, Cicoira M, McCullough PA. Cardiorenal syndrome type 1: pathophysiological crosstalk leading to combined heart and kidney dysfunction in acutely decompensated heart failure[J]. J Am Coll Cardiol. 2012;60(12):1031–42. 22840531 10.1016/j.jacc.2012.01.077

[48] Zhang LE, Yang CL. Impact of serum creatinine level on prognosis of STEMI patients[J]. Chin J Front Med (Electron Ed). 2019;11(8):98–101.

[49] Savage ML, Hay K, Anderson B et al. The Prognostic Value of Echocardiographic Wall Motion Score Index in ST-Segment Elevation Myocardial Infarction[J]. Crit Care Res Pract. 2022, 2022: 1–9. 10.1155/2022/8343789. 10.1155/2022/8343785 PMC9671736 36405398

[50] Playford D, Stewart S, Harris SA, et al. Pattern and prognostic impact of regional wall motion abnormalities in 255,697 men and 236,641 women investigated with Echocardiography[J]. J Am Heart Assoc. 2023;12(22):e031243. 37947119 10.1161/JAHA.123.031243 PMC10727298

[51] Espersen C, Modin D, Platz E, et al. Global and regional wall motion abnormalities and incident heart failure in the general population[J]. Int J Cardiol. 2022;357:146–51. 35304187 10.1016/j.ijcard.2022.03.027

[52] Guo C, Gao B, Han X, et al. Interpretable artificial intelligence model for predicting heart failure severity after AMI[J]. BMC Cardiovasc Disord. 2025;25(1):1–12. 40355836 10.1186/s12872-025-04818-1 PMC12067671

[53] Wang JX, Li CP, Cui Z, et al. Machine learning algorithms to predict HFpEF among patients with premature myocardial infarction[J]. Front Cardiovasc Med. 2025;12:1571185. 40401222 10.3389/fcvm.2025.1571185 PMC12092383

[54] Lin Q, Zhao W, Zhang H, et al. Predicting the risk of heart failure after AMI using an interpretable machine learning model[J]. Front Cardiovasc Med. 2025. 10.3389/fcvm.2025.1444323. 10.3389/fcvm.2025.1444323 PMC11802525 39925976

