# Supplementary material for: Systematic review and meta - analysis of risk prediction models for heart failure after PCI in patients with acute myocardial infarction
Source: BMC Cardiovasc Disord. 2026 Jan 5;26:105. doi: 10.1186/s12872-025-05406-z (PMC12870084; doi:10.1186/s12872-025-05406-z)

1.Killip分类


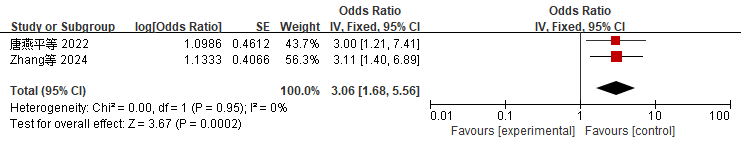


22.年龄


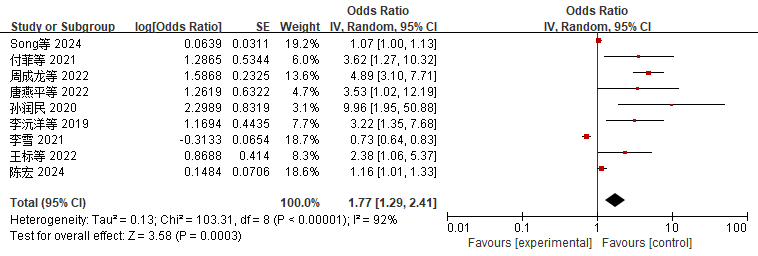


3.Gensini评分


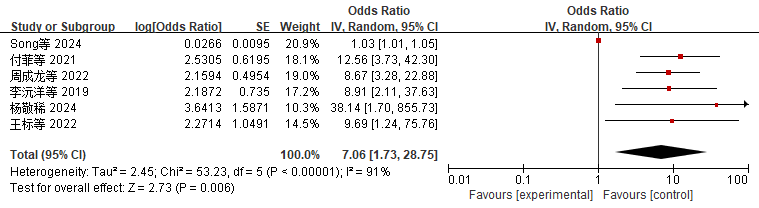


4.LVEF


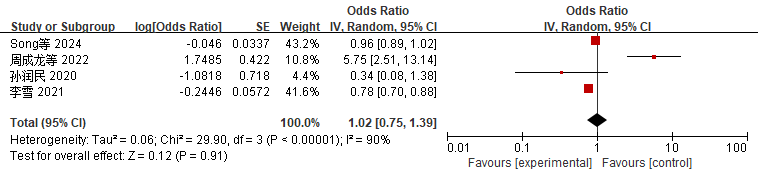


5.D-二聚体水平


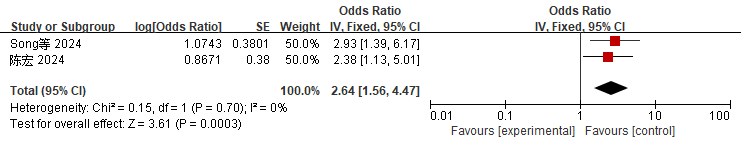


6.NT-proBNP


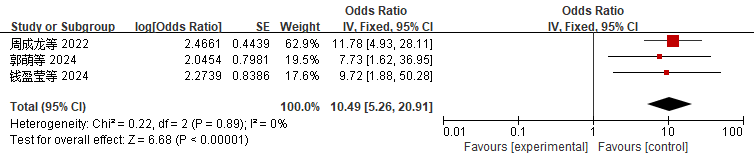


7.NHR


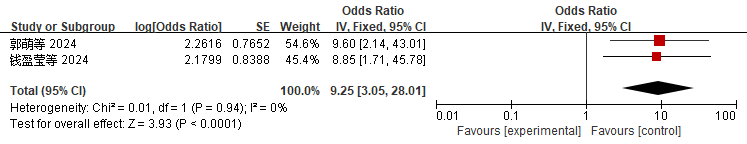


8.NLR


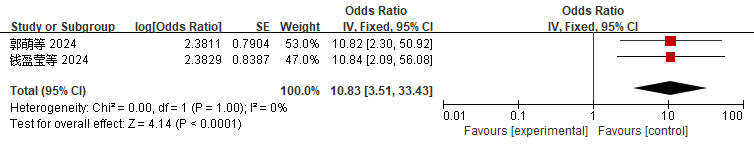


9.MSI


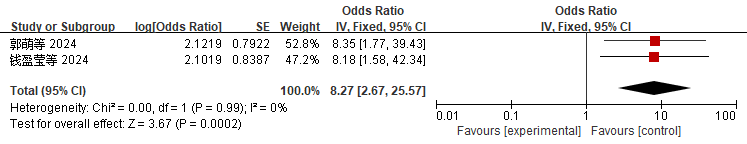


10.肌钙蛋白I


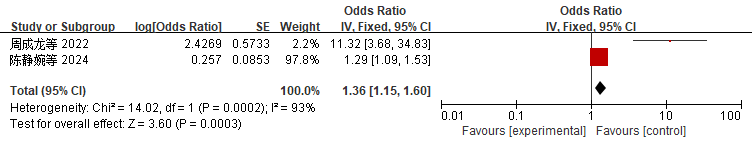


11.心肌肌钙蛋白T


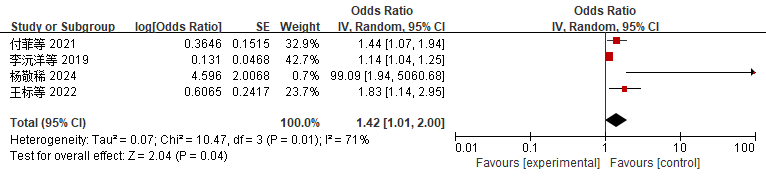


12.血清肌酐


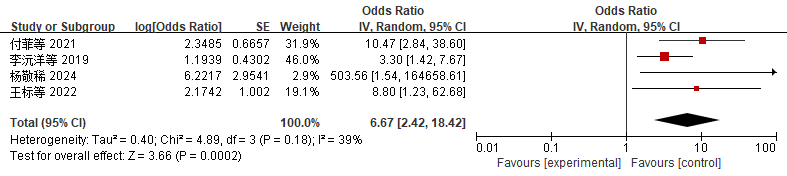


13.hs-CRP


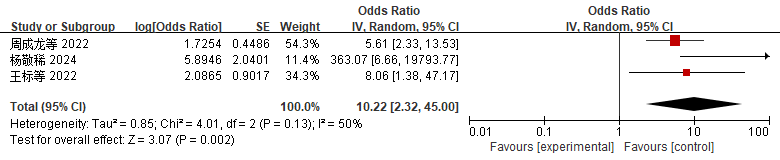


14.室壁运动幅度


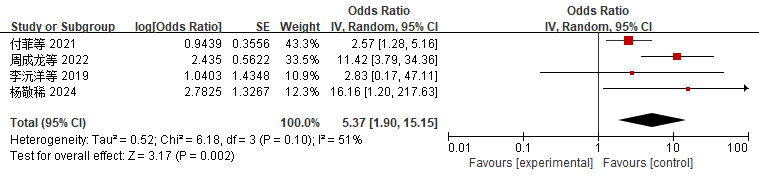


15.高血压


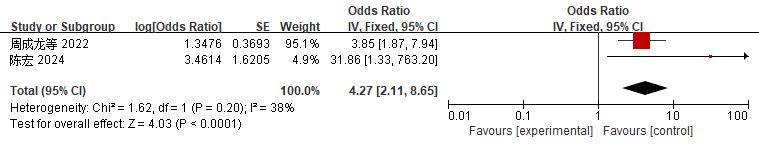


16.发病至就诊时间


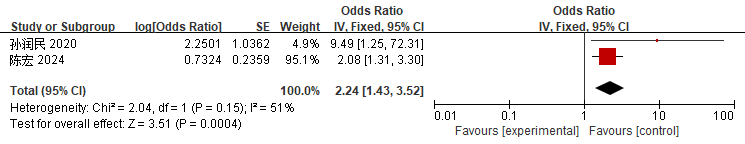


17.病变血管支数


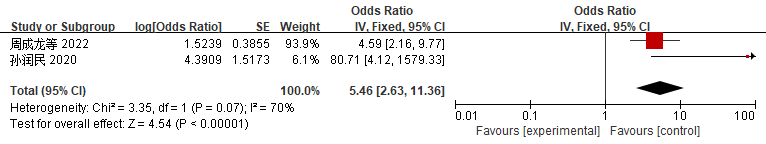


18.糖尿病


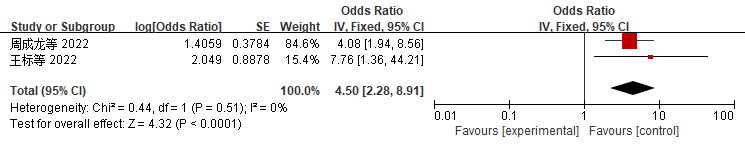


19.心律失常


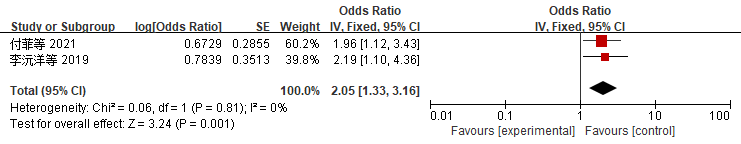


20.心脏结构改变


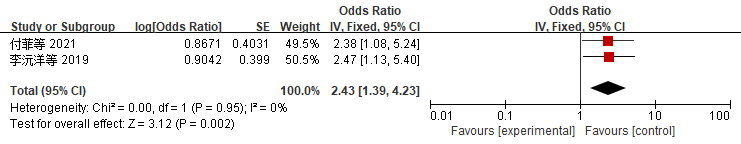

Supplement: Supplementary file 1 — Supplementary Material 1. [file 12872_2025_5406_MOESM1_ESM.docx]
