# Supplementary material for: Systematic review and meta - analysis of risk prediction models for heart failure after PCI in patients with acute myocardial infarction
Source: BMC Cardiovasc Disord. 2026 Jan 5;26:105. doi: 10.1186/s12872-025-05406-z (PMC12870084; doi:10.1186/s12872-025-05406-z)
Supplement: Supplementary file 2 — Supplementary Material 2. [file 12872_2025_5406_MOESM2_ESM.docx]

Table 1-1 Definitions of Heart Failure and Follow-up

| Literature | Definition of heart failure | follow-up |
| --- | --- | --- |
| Zhang 2024^[13]^ | The primary outcome metric focused on the emergence of heart failure symptoms during the patient’s hospital stay. This was clinically defined by the onset of new symptomatic manifestations, clinical signs, and pertinent diagnostic changes, indicating heart failure. | 6 months |
| Song 2024^[14]^ | The diagnosis of HF was made according to the diagnostic criteria in the Chinese guidelines for the management of acute HF in the emergency setting (2022). | in-hospital |
| Guo 2024^[15]^ | Heart failure is defined according to the 2022 ESC Guidelines for the Diagnosis and Treatment of Acute and Chronic Heart Failure. It is diagnosed when echocardiography reveals a left ventricular ejection fraction (LVEF) < 50%, accompanied by clinical manifestations including elevated jugular venous pressure, cardiomegaly, easy fatigability, ankle/lower extremity edema, reduced exercise tolerance, dyspnea, and positive hepatojugular reflux sign. | in-hospital |
| Qian 2024^[16]^ | Heart failure in hospitalized patients was evaluated with reference to the 2021 ESC Guidelines for the Diagnosis and Treatment of Acute and Chronic Heart Failure. It was defined as echocardiographic evidence of left ventricular ejection fraction (LVEF) < 50%, combined with clinical manifestations including reduced exercise tolerance, dyspnea, easy fatigability, ankle or lower extremity edema, and/or accompanied by pulmonary congestion, elevated jugular venous pressure, cardiomegaly, and positive hepatojugular reflux sign. | in-hospital |
| Chen 2024^[17]^ | Left ventricular ejection fraction (LVEF) < 50% is defined as heart failure. | 6 months |
| Yang 2024^[18]^ | Assessment and diagnosis were performed in accordance with the *2016 ESC Guidelines for the Diagnosis and Treatment of Acute and Chronic Heart Failure*. | 6 months |
| Chen 2024^[19]^ | Heart failure was diagnosed with reference to the *2020 Chinese Expert Consensus on the Prevention and Treatment of Heart Failure After Myocardial Infarction*. | in-hospital |
| Zhou 2022^[20]^ | Patients presented with clinical symptoms and signs including tachypnea, increased heart rate, paroxysmal nocturnal dyspnea, dyspnea on exertion, edema, significant reduction in exercise tolerance, and pulmonary rales. They had a Killip classification of cardiac function ≥ Grade 2 and were confirmed by examinations such as electrocardiogram, echocardiography, and relevant laboratory indicators. | 12 months |
| Tang 2022^[21]^ | In the *Nomenclature and Diagnosis of Ischemic Heart Disease*, the diagnostic criteria for acute myocardial infarction complicated with heart failure are consistent with Killip classification of cardiac function Grade Ⅱ~Ⅳ. | 1 month |
| Wang 2022^[22]^ | Heart failure (HF) was diagnosed based on clinical manifestations, combined with examinations including electrocardiogram (ECG), echocardiography, laboratory tests, and biomarkers. The Killip classification of cardiac function was Grade Ⅱ~Ⅳ. | More than 6 months |
| Fu 2021^[23]^ | Heart failure (HF) was diagnosed based on clinical manifestations, as well as examinations including electrocardiogram (ECG), echocardiography, laboratory tests, and biological biomarkers, with the Killip classification of cardiac function being Grade Ⅱ–Ⅳ. | Three years |
| Li 2021^[24]^ | The diagnostic criteria for heart failure refer to the *2020 Chinese Expert Consensus on the Prevention and Treatment of Heart Failure After Myocardial Infarction*. The diagnostic cut-off values of N-terminal pro-brain natriuretic peptide (NT-proBNP) vary by age and renal function as follows: ① > 450 ng/L for patients < 50 years old; ② > 900 ng/L for patients ≥ 50 years old; ③ > 1800 ng/L for patients 75 years old; ④ > 1200 ng/L for patients with estimated glomerular filtration rate (eGFR) < 60 ml/min. The severity was evaluated using the Killip classification of cardiac function. | in-hospital |
| Sun 2020^[25]^ | Heart failure was diagnosed with reference to the method of the New York Heart Association (NYHA). | 1 month |
| Li 2019^[26]^ | Heart failure (HF) is a syndrome dominated by circulatory dysfunction, resulting from diastolic and/or systolic myocardial dysfunction that leads to insufficient cardiac output to meet the metabolic needs of tissues. | Three years |
