# Supplementary material for: Systematic review and meta - analysis of risk prediction models for heart failure after PCI in patients with acute myocardial infarction
Source: BMC Cardiovasc Disord. 2026 Jan 5;26:105. doi: 10.1186/s12872-025-05406-z (PMC12870084; doi:10.1186/s12872-025-05406-z)
Supplement: Supplementary file 3 — Supplementary Material 3. [file 12872_2025_5406_MOESM3_ESM.docx]

**第一部分：偏倚风险评价**

| **1研究对象** | **□低偏倚风险 □高偏倚风险 □不清楚** |
| --- | --- |
| 1.1是否使用了合适的数据来源，例如队列、随机对照或巢式病例-对照研究数据? | □是/可能是  □不是/可能不是  □没有信息 |
| 1.2所有研究对象的纳入和排除标准是否合适? | □是/可能是  □不是/可能不是  □没有信息 |
| **2预测因子** | **□低偏倚风险 □高偏倚风险 □不清楚** |
| 2.1对所有研究对象而言，是否都以相同的方式定义和评估预测变量? | □是/可能是  □不是/可能不是  □没有信息 |
| 2.2是否在不了解结局指标的情况下进行预测变量评估? | □是/可能是  □不是/可能不是  □没有信息 |
| 2.3在应用模型时，是否可以得到所有的预测变量信息? | □是/可能是  □不是/可能不是  □没有信息 |
| **3结果** | **□低偏倚风险 □高偏倚风险 □不清楚** |
| 3.1结局的定义是否合适? | □是/可能是  □不是/可能不是  □没有信息 |
| 3.2结局是否使用预先制定或标准的定义? | □是/可能是  □不是/可能不是  □没有信息 |
| 3.3结局的定义是否排除了预测变量? | □是/可能是  □不是/可能不是  □没有信息 |
| 3.4对所有研究对象而言，是否都以相同的方式定义和确定结局? | □是/可能是  □不是/可能不是  □没有信息 |
| 3.5是否在不了解预测变量的情况下确定结局? | □是/可能是  □不是/可能不是  □没有信息 |
| 3.6预测因子评估和结局确定之间的时间间隔是否合适? | □是/可能是  □不是/可能不是  □没有信息 |
| **4统计分析** | **□低偏倚风险 □高偏倚风险 □不清楚** |
| 4.1样本量是否合理? | □是/可能是  □不是/可能不是  □没有信息 |
| 4.2对连续和分类预测变量处理是否合适? | □是/可能是  □不是/可能不是  □没有信息 |
| 4.3所有纳入的研究对象是否均进行统计分析? | □是/可能是  □不是/可能不是  □没有信息 |
| 4.4缺失数据的研究对象处理是否合适? | □是/可能是  □不是/可能不是  □没有信息 |
| *4.5是否避免基于单变量分析筛选预测变量? | □是/可能是  □不是/可能不是  □没有信息 |
| 4.6数据中的复杂性处理是否恰当? | □是/可能是  □不是/可能不是  □没有信息 |
| 4.7对模型的相关性能指标的评估是否合理? | □是/可能是  □不是/可能不是  □没有信息 |
| *4.8是否将模型性能中的模型过拟合和拟合不足考虑在内? | □是/可能是  □不是/可能不是  □没有信息 |
| *4.9最终模型中的预测变量及其分配权重是否与报告的多变量分析结果一致? | □是/可能是  □不是/可能不是  □没有信息 |

***：该条目仅针对预测模型开发研究；**

**第二部分：适用性评价**

| 1. 研究对象 □ 低适用性风险 □ 高适用性风险 □ 不清楚 |
| --- |
| 低适用性风险：原始研究纳入对象和临床设计与系统评价问题相符。  高适用性风险：原始研究纳入对象和临床设计与系统评价研究问题不同。  不清楚：未报告研究对象和临床设计的相关信息。 |
| 2. 预测因子 □ 低适用性风险 □ 高适用性风险 □ 不清楚 |
| 低适用性风险：原始研究预测因子的定义、评估和评估时间与系统评价问题相符。  高适用性风险：原始研究预测因子的定义、评估和评估时间与系统评价问题不同。  不清楚：未报告预测因子的信息。 |
| 3. 结果 □ 低适用性风险 □ 高适用性风险 □ 不清楚 |
| 低适用性风险：结果的定义、时间间隔和分析方法与系统评价问题相符。  高适用性风险：结果的定义、时间间隔和分析方法是系统评价问题所预期的其它结果。  不清楚：未报告结果的定义、时间间隔和分析方法的信息。 |
| 整体适用性评价：□ 低适用性风险 □ 高适用性风险 □ 不清楚 |
| ★：该条目仅限于模型开发研究；  EPV：每个自变量的事件数（events per variable），指研究对象中较少组的数量除以自变量的个数；  区分度：是指模型区分是否患有待诊断的疾病（诊断模型）或是否发生预期的事件（预后模型）的能力，也就是将患者按照风险的大小进行排序的能力；  校准度：评估预测的概率与实际观察到的概率的一致性。 |
