# Supplementary material for: Systematic review and meta - analysis of risk prediction models for heart failure after PCI in patients with acute myocardial infarction
Source: BMC Cardiovasc Disord. 2026 Jan 5;26:105. doi: 10.1186/s12872-025-05406-z (PMC12870084; doi:10.1186/s12872-025-05406-z)
Supplement: Supplementary file 4 — Supplementary Material 4. [file 12872_2025_5406_MOESM4_ESM.docx]

**Search Strategy：**

**1.PubMed**

#1 acute anterior wall myocardial infarction"[MeSH] OR "acute inferior myocardial infarction"[MeSH] OR "AMI"[Title/Abstract] OR "myocardial infarction"[Title/Abstract] OR "acute myocardial infarction"[Title/Abstract] OR "AMI"[Title/Abstract] OR "acute MI"[Title/Abstract] OR "STEMI"[Title/Abstract] OR "NSTEMI"[Title/Abstract]

#2 "percutaneous coronary intervention"[MeSH] OR "PCI"[Title/Abstract] OR "percutaneous coronary intervention"[Title/Abstract] OR "post-PCI"[Title/Abstract] OR "coronary angioplasty"[Title/Abstract] OR "stenting"[Title/Abstract]

#3 "heart failure"[Mesh] OR "Heart Failure"[Title/Abstract] OR "HF"[Title/Abstract]

#4 "risk assessment"[Mesh] OR "models, statistical"[Mesh] OR "risk prediction model"[Title/Abstract] OR "predict"[Title/Abstract] OR "prediction model"[Title/Abstract] OR "risk prediction"[Title/Abstract] OR "risk assessment"[Title/Abstract] OR "risk evaluation"[Title/Abstract] OR "risk score"[Title/Abstract] OR "risk stratification model"[Title/Abstract] OR "prediction tool"[Title/Abstract] OR "prognostic model"[Title/Abstract] OR "stratification model"[Title/Abstract] OR "prediction rule"[Title/Abstract] OR "forecast model"[Title/Abstract]

#5 #1 AND #2 AND #3 AND #4

**2.Web of Science**

#1 TS=("acute myocardial infarction" OR "AMI" OR "myocardial infarction")

#2 TS=("percutaneous coronary intervention" OR "PCI" OR "angioplasty" OR "stenting")

#3 TS=("heart failure" OR "cardiac failure" OR "HF")

#4 TS=("risk prediction model" OR "prediction model" OR "prognostic model" OR "risk score" OR "prediction rule" OR "machine learning model" OR "artificial intelligence" OR "deep learning" OR "predict" OR "prediction tool")

#5 #1 AND #2 AND #3 AND #4

**3.Embase**

ab,ti=("myocardial infarction" ) AND ("risk assessment" OR "prediction model" OR "risk prediction model" OR "prediction model" OR "prognostic mode" OR "risk score" OR "prediction rule") AND ("heart failure")

**4.Cochrane Library**

#1 MeSH descriptor: [Myocardial Infarction] explode all trees

#2 MeSH descriptor: [Percutaneous Coronary Intervention] explode all trees

#3 MeSH descriptor: [Heart Failure] explode all trees

#4 MeSH descriptor: [Risk Assessment] explode all trees

#5 #1 AND #2 AND #3 AND #4

#6 "acute myocardial infarction":ti,ab,kw AND "PCI":ti,ab,kw AND "heart failure":ti,ab,kw AND "predict":ti,ab,kw)

#7 #5 OR #6

**5.China Knowledge Network**

#1 SU=('急性心肌梗死' OR '急性心梗' OR 'AMI')

#2 SU=('PCI' OR '经皮冠状动脉介入治疗' OR '冠状动脉介入' OR '血管成形术')

#3 SU=('心力衰竭' OR '心衰' OR 'HF')

#4 SU=('模型' OR '预测' OR '风险' OR '评分' OR '列线图' OR '机器学习模型' OR '预测规则')

#5 #1 AND #2 AND #3 AND #4

**6.VIP network**

#1 M=('急性心肌梗死' OR '急性心梗' OR 'AMI')

#2 M=('PCI' OR '经皮冠状动脉介入治疗' OR '冠状动脉介入')

#3 M=('心力衰竭' OR '心衰' OR 'HF')

#4 M=('模型' OR '预测' OR '风险' OR '评分' OR '列线图' OR '机器学习模型' OR '预测规则')

#5 #1 AND #2 AND #3 AND #4

**7.Wanfang Data knowledge service platform**

SU=((("急性心肌梗死" OR "心肌梗死" OR AMI) AND

("PCI" OR "经皮冠状动脉介入治疗" OR "冠状动脉介入") AND

("心力衰竭" OR "心衰" OR "HF") AND

("风险预测模型" OR "预测模型" OR "预后模型" OR "风险评分" OR "列线图" OR "预测规则" OR "机器学习模型")))

**8.Chinese medical journal full-text database**

TM=("急性心肌梗死") AND ("心力衰竭")

**9.China Biomedical Literature Database**

#1 "急性心肌梗死"[常用字段] OR "急性心梗"[常用字段] OR "AMI"[常用字段]

#2 "PCI"[常用字段] OR "经皮冠状动脉介入治疗"[常用字段] OR "冠状动脉介入"[常用字段]

#3 "心力衰竭"[常用字段] OR "心衰"[常用字段] OR "HF"[常用字段]

#4 "模型"[常用字段] OR "预测"[常用字段] OR "风险"[常用字段] OR "评分"[常用字段] OR "列线图"[常用字段] OR "机器学习模型"[常用字段] OR "预测规则"[常用字段]

#5 #1 AND #2 AND #3 AND #4
