# Supplementary figures and images for: Systematic review and meta - analysis of risk prediction models for heart failure after PCI in patients with acute myocardial infarction
Source: BMC Cardiovasc Disord. 2026 Jan 5;26:105. doi: 10.1186/s12872-025-05406-z (PMC12870084; doi:10.1186/s12872-025-05406-z)

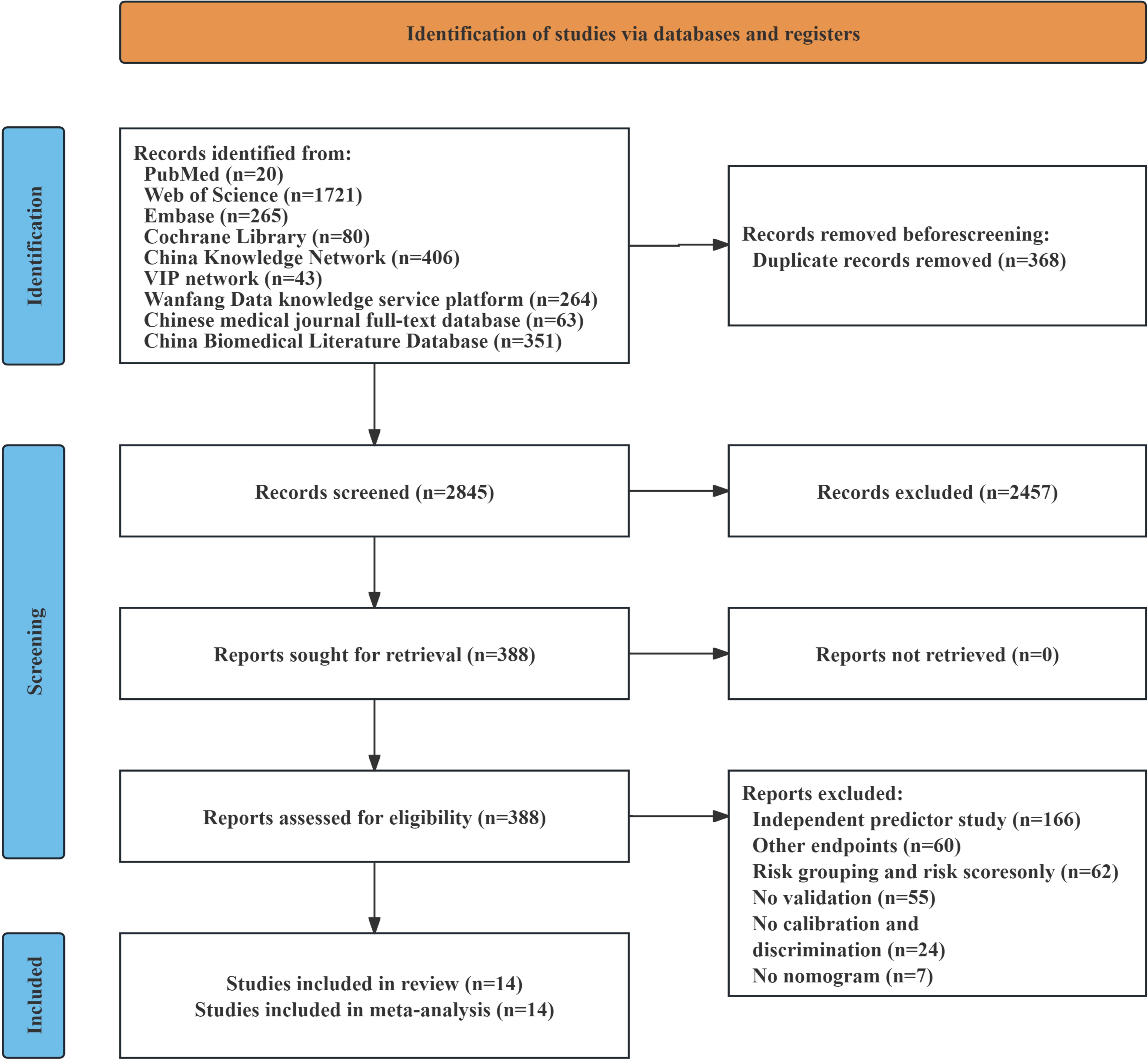

Figure 1 Literature Screening Flowchart

Supplement: Supplementary file 5 — Supplementary Material 5. [file 12872_2025_5406_MOESM5_ESM.pdf]
